# Variation of the T‐wave peak‐end interval and heart rate variability values in healthy males and females at various hours of the same day, and relationship of them

**DOI:** 10.1002/joa3.12296

**Published:** 2020-01-08

**Authors:** Ayhan Cosgun, Huseyin Oren

**Affiliations:** ^1^ Department of Cardiology Sincan State Hospital Ankara Turkey; ^2^ Department of Cardiology Ankara City Hospital Ankara Turkey

**Keywords:** daily variation, heart rate variability, Tp‐e interval, Tp‐e/QTc, Tp‐e/QT

## Abstract

**Background:**

The prolongation of repolarization time between the myocardial epicardium and endocardial cells is closely related to malignant ventricular arrhythmias. The purpose of our study was to compare repolarization markers, namely, T‐wave peak‐end interval (Tp‐e), QT, corrected QT (QTc), Tp‐e/QT, Tp‐e/corrected QT (QTc), and Heart Rate Variability (HRV) values in healthy men and women and to investigate their daily variations.

**Methods:**

A total of 74 male and 78 female participants, being a government employee, and having no health problems, were included in the two study groups (males and females). A 24‐hour, 12‐lead Holter monitoring was performed on the volunteers. Then, the Tp‐e interval and QT interval were measured on recordings. cTp‐e and QTc were calculated by the use of Bazzet's formula.

**Results:**

There was no statistically significant difference between the groups in the cTp‐e interval at 07.00 pm; however, it was significantly lower in the female group as compared with the male group at 07.00 am and 01.00 pm. It was significantly higher in the female group at 01.00 am compared with the male group. There were statistically significant moderate negative correlations between Tp‐e intervals and a standard deviation of between two normal beats interval (SDNN) values at various hours of the same day.

**Conclusion:**

There were statistically significant differences in terms of Tp‐e and cTp‐e intervals at various hours of the same day in both groups. In addition, there were statistically significant moderate negative correlations between Tp‐e intervals and SDNN at various hours of the same day.

## Graphical abstract

Malign ventricular arrhythmia is observed mostly in individuals with heart disease, but it may also occur in individuals without underlying heart disease. Ventricular arrhythmias may occur more frequently at certain times. For example, it can follow an increasing trend from Saturday to Monday, while decreasing trend from Monday to Friday. Furthermore, Ventricular arrhythmias can occur at different times on the same day. It can become more frequent and decrease at certain times of the day. Men also have a higher incidence of SCD than women. Therefore, Tp‐e interval, cTp‐e interval, Tp‐e/ QT and Tp‐e/ QTc ratios, which are ventricular repolarization, and Heart Rate Variability Values may vary in healthy men and women at different times of the day.

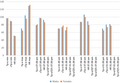

**Abbreviations**
BMI: Body Mass Index
BP: Blood Pressure
COPD: Chronic Obstructive Pulmonary Disease
cTp‐e: Corrected T peak‐end
CVE: Cerebrovascular Event
ECG: Electrocardiography
LDL: Low‐Density Lipoprotein
LF: Absolute power of the low‐frequency band (0.04‐0.15 Hz)
LF/HF: Ratio of LF‐to‐HF power
LV: Left ventricle
pNN50: Percentage of successive RR intervals that differ by more than 50 ms
QTc: Corrected QT
RMSSD: Root mean square of successive RR interval differences
SCD: Sudden Cardiac Death
SDANN Index: Standard deviation of the average NN intervals for each 5 min segment of a 24 h heart rate variability recording
SDNN Index: Mean of the standard deviations of all the NN intervals for each 5 min segment of a 24 h heart rate variability recording
SDNN: Standard Deviation of all NN intervals
TC: Total Cholesterol
Tp‐e: T peak‐end
TSH: Thyroid Stimulating Hormone
TTE: Transthoracic Echocardiography
VLF: Absolute power of the very‐low‐frequency band (0.0033‐0.04 Hz)

## 1 INTRODUCTION

Cardiovascular system‐related deaths are still the most common cause of death in both men and women worldwide. However, there are major differences between men and women. For example, CVS events start in men at an earlier age, and their mortality and morbidity are higher than in women.^1^

Heart rate variability (HRV) is a unit of measurement of the effects of heart‐related functions on the heart of the vagal and sympathetic system. An imbalance or reduction in this unit of measure is closely related to cardiovascular mortality and morbidity.^2^ Impaired autonomic balance against vagal tonus is closely associated with increased cardiovascular mortality and morbidity.^3^ This may help to explain the severity of cardiovascular mortality and morbidity in men and women and the course of events. In some studies, there is a general decrease in HRV with age and deterioration of autonomic balance against vagal tonus, but still more sympathetic tonus in men of the same age group than women.^4^

Many studies show that many mortality and morbid cardiovascular events occur more in the morning than at other times of the day.^5^ Heart rate also follows a certain circadian rhythm. A higher heart rate is detected in both men and women in the daytime compared with nights. However, not only does the heart rate follow a circadian rhythm, but also a circadian rhythm in HRV.^6^ Moreover, HR and HRV were also correlated with hormones secreted by daily circadian rhythms, such as cortisol and melatonin.^7^ Therefore, changes in autonomic cardiovascular regulation during the day can help explain the occurrence of morbid and mortal cardiovascular events at different rates and amounts throughout the day.

It is well known that the electrophysiology of men and women is very different. And it has been proven that women have longer rate‐corrected ventricular repolarization intervals and show a different repolarization pattern than men.^8^ At the same time, in some studies conducted with animal experiments, ventricular repolarization parameters J‐T end and T peak‐end interval times were different in male and female rabbits. This finding may help to explain the different rates and amounts of SCD or malignant ventricular arrhythmias in men and women.^9^

The T wave typically shows the repolarization of myocardial M cells. The T wave is as a result of complex myocardial repolarization including the difference between right and left ventricular repolarization and the gradient between the left ventricular apex and the base.^10^

In general, simple manual methods of T‐wave measurements can be used for cardiovascular risk stratification both in patients with underlying structural heart disease and in the general healthy population.^11^ Therefore, the investigation of T‐wave morphology by simple methods has attracted much attention in the literature. In this regard, a number of simple methods have been developed for the duration, area, amplitude, and slope of the T wave, and this has attracted attention.^12^ Today, one of the most widely used simple methods is the T peak‐end interval (Tp‐e), the distance from the peak of the T wave to the isoelectric line. Significant prolongations in Tp‐e (regional cardiac repolarization dispersion indicator) interval was closely associated with increased cardiovascular deaths.^13^ Human studies for the Tp‐e interval revealed that the Tp‐e interval was not RR interval dependent on age and gender.^14^ In studies on horses, it was found that prolongation of the Tp‐e interval occurred when the heart rate fell, but it has not yet been proven to be associated with cardiovascular mortality.^15^ In another study conducted in horses, it was found that the Tp‐e interval did not change significantly during the day and was independent of RR interval.^16^ However, no study has been conducted to examine the changes in repolarization markers such as Tp‐e, QT, QTc, Tp‐e/QT, Tp‐e/QTc, and HRV parameters in healthy men and women. The aim of our study was to investigate the changes in these parameters in healthy male and female individuals at different times during the same day.

## 2 METHODS

### 2.1 Study population

A total of 74 male participants with a mean age of 45.17 ± 4.96 years (Male group) and 78 female participants with a mean age of 47.35 ± 10.84 years (Female group) were included in the study. Three consecutive blood pressure measurements were taken for both groups after a 5‐min rest, and the mean value of these readings was recorded. Participants with mean blood pressure higher than 149/90 mm Hg, a previous diagnosis of hypertension, and those on any antihypertensive medication were excluded from the study. Arterial blood pressures of the patients were recorded, and resting electrocardiograms (ECG) were acquired at 25 mm/sec speed and 10 mm/mV amplitude. The participants were then taken for routine physical examination during which those with smoking history, with findings that may impact the electrophysiology of the heart, and those with a body mass index (BMI) higher than 30 kg/m^2^ were excluded from the study. After the routine physical examination, routine blood tests and transthoracic echocardiograms (TTE) were performed. In routine blood tests, participants with anemia; poor thyroid, liver, or kidney function; antidepressant medication history, electrolyte disorder, white blood cell count >10,000/dl; patients diagnosed with cancer or receiving chemotherapy, patients with past cerebrovascular event, chronic obstructive pulmonary disease history, and those receiving bronchodilator treatment; patients who had coronary angiography and receiving any treatment, and patients with significant valvular heart disease, left ventricular hypertrophy, and systolic pulmonary arterial pressure >25 mm Hg in TTE were also excluded from the study. We did not find any statistically significant difference in terms of sociodemographic and basic clinical characteristics between the two groups (Table 1).

**Table 1:** The sociodemographic and basal clinic values of the groups

Variables	Male group (n = 74)	Female group (n = 78)	T‐value	*P*‐value
Age, years	45.17 ± 4.96	45.58 ± 7.28	–0.40	.34
BMI, kg/m^2^	25.98 ± 3.2	24.56 ± 3.1	0.56	.28
BP, mm Hg	124.85 ± 10.42	123.91 ± 11.3	0.58	.27
LV mass, gr	173.26 ± 28.3	169.37 ± 25.69	0.93	.17
Hemoglobin, gr/dL	14.4 ± 1.4	13.56 ± 1.6	0.92	.17
Basal sPAP, mm Hg	15.56 ± 3.4	14.78 ± 4.1	0.85	.19
TC. mg/dL	192.81 ± 37.46	186.75 ± 25.43	1.26	.10
LDL, mg/dL	137.75 ± 18.13	134.51 ± 15.33	0.91	.18
Triglyceride, mg/dL	175.48 ± 33.48	166.35 ± 29.95	0.75	.22
Calcium, mg/dL	9.2 ± 0.6	9.1 ± 0.8	0.05	.47
Sodium, mEq/L	141.45 ± 3.4	139.4 ± 4.1	0.76	.22
TSH, mIU/L	3.4 ± 1.2	3.1 ± 1.0	0.14	.44

Abbreviations: BMI, body mass index; BP, blood pressure; LDL, low‐density lipoprotein; LV, left ventricle; TC, total cholesterol; TSH, thyroid stimulating hormone.

### 2.2 Electrocardiography

The 12‐lead ECG recordings were obtained from 24‐hour Holter recordings. The ECG length was 10 seconds; therefore, depending on the heart rate, there were 4 to 6 beats per lead. ECGs were measured manually with the use of a magnifying glass (TorQ; 150 mm Digital Caliper LCD) by two blinded cardiologists. The RR interval, QT interval, and T peak‐end interval of the ECG recordings obtained from 24‐hour Holter monitorization at 07.00 am, 01.00 pm, 07.00 pm, and 01.00 am were measured manually with a 0.01 mm accuracy.

The RR interval was recorded as the time from the peak of an R wave to the peak point of the next R wave. This measurement was calculated at least in three leads and between at least three consecutive R waves. The QT interval was accepted as the point where the T wave returned to the isoelectric line from the onset of the QRS wave. This measurement was calculated at least in nine leads and three consecutive QRS waves in one lead. The data of ECG with papers at a speed of 25 mm/sec and amplitude of 10 mm/mV, measured in mm with the digital caliper, were calculated as millisecond multiplied by 40. The QTc was calculated using Bazett's formula.

Tp‐e interval was calculated by including the T wave from the peak to the baseline in the V_2__–__5_ leads. The Tp‐e interval was calculated as a millisecond with the measured value in millimeter by multiplying 40. If the U wave was present, the T wave end was defined as the nadir between the T and U waves. The cTp‐e interval was calculated using Bazett's formula, and the arithmetic averages of measurements were used for analysis.

### 2.3 Twenty‐hour 12‐lead rhythm Holter

A 12‐lead Holter (DMS‐300, 12L, serial number: 8438, DM Software, Vers:11.4.0054a) monitor was connected to the patients at 02.00 pm and removed at 02.00 pm the next day. A 24‐hour detailed evaluation was performed for assessing the 24‐hour rhythm Holter. During the evaluation, patients having the right or left bundle branch block, patients detected with more than 200/day atrial or ventricular extra‐systoles, those having irregular or persistent supraventricular or ventricular tachycardia, patients with ST‐T wave variations in ECG screening, and those with less than 22 hours of Holter recording were excluded from the study. ECG records including minimum and maximum heart rates of patients were acquired and calculations were made. In addition, ECG and SDNN values of the patients were recorded at 07.00 pm, 01.00 am, 07.00 am, and 01.00 pm, followed by appropriate calculations. And Heart Rate Variability values were recorded and compared for each group. SDNN (Standard Deviation of all NN intervals), SDANN Index (Standard deviation of the average NN intervals for each 5 minutes segment of a 24 hours heart rate variability recording), SDNN Index (Mean of the standard deviations of all the NN intervals for each 5 minutes segment of a 24 hours heart rate variability recording), RMSSD (Root mean square of successive RR interval differences), pNN50 (Percentage of successive RR intervals that differ by more than 50 ms), VLF (Absolute power of the very‐low‐frequency band [0.0033‐0.04 H]), LF (Absolute power of the low‐frequency band [0.04‐0.15 Hz]), and LF/HF (Ratio of LF‐to‐HF power) values were recorded and compared between two groups.

### 2.4 Statistical analysis

Continuous variables were expressed as a mean ± standard deviation. Categorical variables were expressed as percentages. Student's t test and the Chi‐square test were used for the comparison of continuous and categorical variables, respectively. For repeated variables, one‐way analysis of variance (ANOVA) for repeated measures was used. Pearson's correlation coefficient was used to examine the correlation between the two variables. A *P* < .05 was considered to be statistically significant. Statistical analysis was performed using a commercially available statistical package SPSS version 20.0 (IBM Co.).

### 2.5 Transthoracic echocardiography

All participants underwent two‐dimensional echocardiography examination. We obtained standard parasternal long‐axis, mid‐ventricular short‐axis, apical long‐axis, apical 2‐ and 4‐chamber images using the Philips HD11XE, 2012 Netherlands.

### 2.6 Ethical approval

All procedures performed in studies involving human participants were in accordance with the ethical standards of the Turkey Research Committee and with the 1964 Helsinki declaration and its later comparable ethical standards. Written permission was obtained from the Hospital Management Committee. The informed patient consent was obtained from each subject.

## 3 RESULTS

There was no statistically significant difference between the male and female groups in terms of sociodemographic and basal clinical findings (Table 1, *P* > .05).

A comparison of male and female groups revealed no statistically significant difference in terms of Tp‐e and cTp‐e intervals at a minimum heart rate (HR), Tp‐e and cTp‐e intervals at 07.00 pm, and Tp‐e intervals at 01.00 am and 07.00 am. However, Tp‐e at maximum HR, cTp‐e at maximum HR, HR at 07.00 pm, 07.00 am, and 01.00 pm, cTp‐e intervals at 07.00 am and 01.00 pm, and Tp‐e interval at 01.00 pm were statistically significantly higher in males than females. In addition, the females had significantly higher cTp‐e and HR at 01.00 am when compared with that in the males (Table 2).

**Table 2:** The comparison of the 12L‐Holter ECG values between the groups, part 1

Variables	Male group	Female group	T‐value	*p*‐value
Tp‐e min., ms	97.13 ± 10.22	97.05 ± 13.64	0.10	.45
HR min., b/min	51.59 ± 6.0	50.56 ± 4.33	0.21	.11
cTp‐e min., ms	89.95 ± 10.9	88.84 ± 11.66	0.58	.27
Tp‐e max., ms	70.41 ± 5.42	65.15 ± 9.56	4.14	<.01
HR max., b/min	129.46 ± 6.88	131.83 ± 6.46	2.34	.01
cTp‐e max., b/min	104.37 ± 7.04	95.61 ± 13.4	5.00	<.01
Tp‐e 07.00 pm, ms	78.75 ± 10.57	81.12 ± 7.22	1.62	.051
HR 07.00 pm, b/mn	93.01 ± 8.61	86.46 ± 9.66	4.09	<.01
cTp‐e 07.00 pm, ms	98.04 ± 10.8	97.37 ± 1.26	0.75	.22
Tp‐e/QT 07.00 pm	0.211 ± 0.02	0.223 ± 0.02	−3.69	<.01
Tp‐e/QTc 07.00 pm	0.170 ± 0.02	0.186 ± 0.02	−4.93	<.01
Tp‐e 01.00 am, ms	70.32 ± 8.97	70.05 ± 6.74	021	.41
HR 01.00 am, b/mn	65.56 ± 5.35	74.03 ± 8.6	–7.21	<.01
cTp‐e 01.00 am, ms	74.08 ± 8.72	77.91 ± 7.04	–2.98	<.01
Tp‐e/QT 01.00 am	0.174 ± 0.02	0.182 ± 0.01	−2.46	.01
Tp‐e/QTc 01.00 am	0.167 ± 0.02	0.165 ± 0.02	0.61	.53
Tp‐e 07.00 am, ms	87.64 ± 12.67	87.34 ± 9.22	0.16	.43
HR 07.00 am, b/mn	89.06 ± 7.92	79.41 ± 9.98	6.58	<.01
cTp‐e 07.00 am, ms	106.55 ± 15.56	100.21 ± 12.11	2.80	<.01
Tp‐e/QT 07.00 am	0.232 ± 0.03	0.224 ± 0.02	1.92	.056
Tp‐e/QTc 07.00 am	0.192 ± 0.03	0.196 ± 0.02	−0.97	.33
Tp‐e 01.00 pm, b/mn	70.05 + 6.61	65.2 ± 6.17	4.67	<.01
HR 01.00 pm, b/mn	81.9 ± 7.16	77.42 ± 8.96	3.39	<.01
cTp‐e 01.00 pm, ms	80.51 ± 12.56	73.76 ± 7.82	3.99	<.01
Tp‐e/QT 01.00 pm	0.185 ± 0.02	0.176 ± 0.01	3.53	<.01
Tp‐e/QTc 01.00 pm	0.158 ± 0.01	0.155 ± 0.01	1.84	.06

Abbreviations: b/min, beat/minute; cTp‐e, corrected T peak‐end; H, heart rate; QT, QT interval; QTc, corrected QT interval; Tp‐e, T peak‐end.

In the male group, SDNN 24‐hour, SDANN Index, SDNN Index, rMSSD, LF, maximum Spectral Power, 24‐hour Spectral Power, and SDNN 01.00 am values were significantly higher than in the female group. There were no differences between the two groups in terms of pNN50, VLF, LF/HF ratio, minimum Spectral Power, maximum QT and minimum QT interval, and SDNN values at 07.00 pm. In addition, in the male group, SDNN at 07.00 am, SDNN at 01.00 pm, and HF values were significantly lower than in the female group (Table 3).

**Table 3:** The comparison of the 12L‐Holter ECG values between the groups, part 2

Variables	Male group	Female group	T‐value	*P*‐value
SDNN 24‐Hour, ms	145.51 ± 41.43	125.33 ± 27.76	3.54	<.01
SDANN Index, ms	133.13 ± 41.51	115.82 ± 27.35	3.05	<.01
SDNN Index, ms	59.24 ± 16.16	51.67 ± 14.59	3.03	<.01
RMSSD, ms	51.13 ± 77.86	26.31 ± 11.47	2.78	<.01
pNN50, %	9.13 ± 7.36	8.05 ± 7.42	0.90	.36
VLF	2365.75 ± 1595.45	2308.11 ± 1524.26	0.22	.82
LF	940.82 ± 758.99	737.74 ± 419.45	2.05	.045
HF	276.65 ± 258.91	382.11 ± 427.55	−1.92	.048
LF/HF	4.11 ± 2.16	3.01 ± 1.65	3.51	<.01
SP‐24 Hour	3766.44 ± 1878.78	2865.31 ± 1679.29	3.12	<.01
Min SP	1226.48 ± 751.25	1081.11 ± 722.16	1.21	.22
Max SP	9339.36 ± 5805.67	6449.61 ± 4158.61	3.54	<.01
Average HR	73.93 ± 8.01	79.55 ± 10.08	−3.79	<.01
Max QT	477.96 ± 40.06	471.77 ± 39.07	0.96	.33
Max QTc	502.58 ± 35.53	503.67 ± 35.26	−0.19	.84
SDNN 07.00 am, ms	89.45 ± 70.01	134.02 ± 59.56	−4.32	<.01
SDNN 01.00 pm, ms	69.91 ± 24.42	78.94 ± 25.07	2.24	.02
SDNN 07.00 pm, ms	78.03 ± 39.33	77.44 ± 25.85	0.11	.91
SDNN 01.00 am, ms	78.12 ± 30.01	66.33 ± 25.61	2.05	.045

Abbreviations: HR, heart rate; LF, absolute power of the low‐frequency band (0.04‐0.15 Hz); LF/HF, ratio of LF‐to‐HF power; pNN50, percentage of successive RR intervals that differ by more than 50 ms; QT, QT interval; QTc, corrected QT interval; RMSSD, root mean square of successive RR interval differences; SDNN, standard deviation of all NN intervals; SDANN Index, standard deviation of the average NN intervals for each 5 min segment of a 24 h heart rate variability recording; SDNN Index, Mean of the standard deviations of all the NN intervals for each 5 min segment of a 24 h heart rate variability recording; VLF, Absolute power of the very‐low‐frequency band (0.0033‐0.04 Hz).

There were statistically significant moderate negative correlations between Tp‐e intervals and SDNNs at 07.00 am, 01.00 pm, 07.00 pm, and 01.00 am in the male group. In addition, in the male group, there were weak negative but statistically significant correlations between cTp‐e intervals and SDNNs at 07.00 am, 01.00 pm, 07.00 pm, and 01.00 am.

There were statistically significant moderate negative correlations between Tp‐e intervals and SDNNs at 07.00 am, 01.00 pm, 07.00 pm, and 01.00 am in the female group. In addition, in the female group, there were weak negative but statistically significant correlations between cTp‐e intervals and SDNNs at 07.00 am, 01.00 pm, 07.00 pm, and 01.00 am (Table 4).

**Table 4:** The correlation between Tp‐e/cTp‐e intervals and SDNN values at 07.00 am, 01.00 pm, 07.00 pm, and 01.00 am in the male and female group

Variable 1	Variable 2	Correlation	*R*‐value	*p*‐value
Male group (n = 74)				
Tp‐e 07.00 am	SDNN 07.00 am	Moderate negative	−.63	<.01
cTp‐e 07.00 am	SDNN 07.00 am	Weak negative	−.44	<.01
Tp‐e 01.00 pm	SDNN 01.00 pm	Moderate negative	−.57	<.01
cTp‐e 01.00 pm	SDNN 01.00 pm	Weak negative	−.36	<.01
Tp‐e 07.00 mm	SDNN 07.00 pm	Moderate negative	−.51	<.01
cTp‐e 07.00 pm	SDNN 07.00 pm	Weak negative	−.34	<.01
Tp‐e 01.00 am	SDNN 01.00 am	Moderate negative	−.59	<.01
cTp‐e 01.00 am	SDNN 01.00 am	Weak negative	−.33	<.01
Female group (n = 78)				
Tp‐e 07.00 am	SDNN 07.00 am	Moderate negative	−.59	<.01
cTp‐e 07.00 am	SDNN 07.00 am	Weak negative	−.42	<.01
Tp‐e 01.00 pm	SDNN 01.00 pm	Moderate negative	−.55	<.01
cTp‐e 01.00 pm	SDNN 01.00 pm	Weak negative	−.39	<.01
Tp‐e 07.00 mm	SDNN 07.00 pm	Moderate negative	−.58	<.01
cTp‐e 07.00 pm	SDNN 07.00 pm	Weak negative	−.36	<.01
Tp‐e 01.00 am	SDNN 01.00 am	Moderate negative	−.63	<.01
cTp‐e 01.00 am	SDNN 01.00 am	Weak negative	−.45	<.01

Abbreviations: cTp‐e, corrected T peak‐end; SDNN, standard deviation of NN intervals for an hour; Tp‐e, T peak‐end.

**Figure 1:**
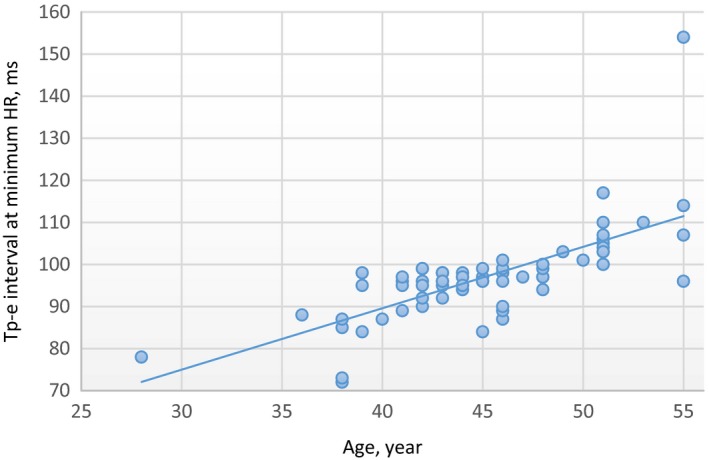
Linear regression graphic of Age and Tp‐e interval at minimum HR in the male group. Abbreviations: Tp‐e, Tpeak‐end interval; HR, heart rate. *R* = .70, *P* < .01, Moderate Positive Correlation. Units of the Measurement: For Tp‐e; millisecond, for HR; beats/minute, for Age; year

**Figure 2:**
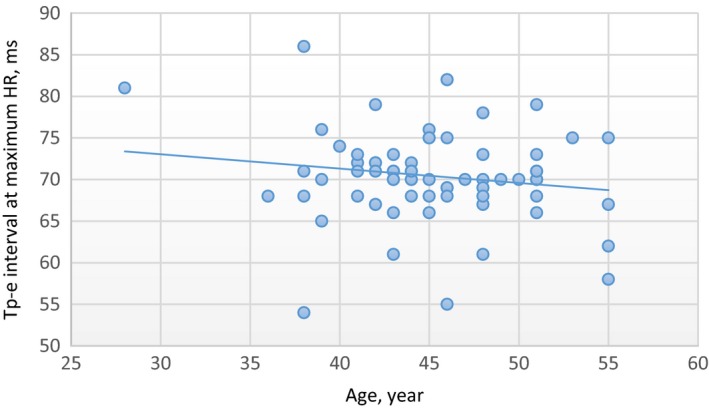
Linear regression graphic of Age and Tp‐e interval at maximum HR in the male group. Abbreviations: Tp‐e, T peak‐end interval; HR, heart rate. *R* = −.15, *P* = .17, No Correlation. Units of the Measurement: For Tp‐e; millisecond, for HR; beats/minute, for Age; year

**Figure 3:**
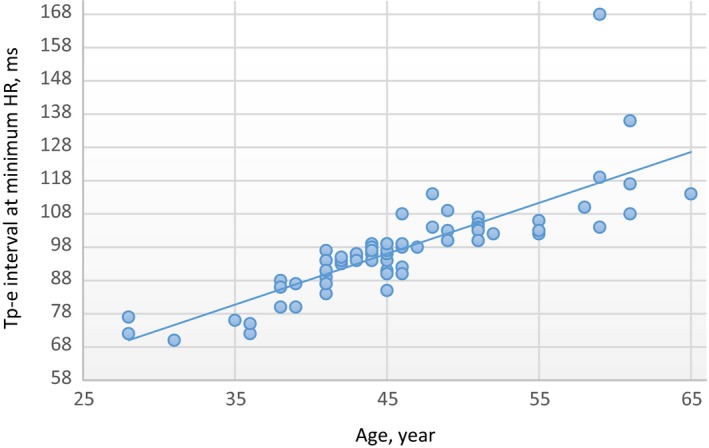
Linear regression graphic of Age and Tp‐e interval at minimum HR in the female group. Abbreviations: Tp‐e, T peak‐end interval; HR, heart rate. *R* = .81, *P* = .01, Strong Positive Correlation. Units of the Measurement: For Tp‐e; millisecond, for HR; beats/minute, for Age; year

**Figure 4:**
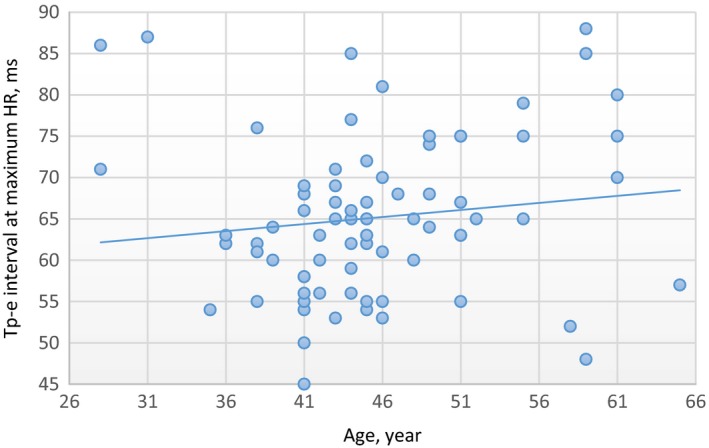
Linear regression graphic of Age and Tp‐e interval at maximum HR in the female group. Abbreviations: Tp‐e, T peak‐end interval; HR, heart rate. *R* = .13, *P* = .25, No Correlation. Units of the Measurement: For Tp‐e; millisecond, for HR; beats/minute, for Age; year

There was a moderate positive correlation at minimum HR between age values and Tp‐e interval values in the male group (*R* = 0.70 and *P* < .01). In addition, there was no correlation at maximum HR between age and Tp‐e interval values in the male group (*R *= −0.15, *P* = .17). In the female group, there was a strong positive correlation at minimum HR between age and Tp‐e interval values (*R* = 0.81 and *P* < .01). In addition, there was no correlation at maximum HR between age and Tp‐e interval values in the female group (*R* = 0.13, *P* = .25) (Figures 1, 2, 3, 4).

## 4 DISCUSSION

In our study, HRV parameters showed significant differences in 24‐hour rhythm Holter analysis. There were also statistically significant differences in cardiac repolarization markers and SDNN between men and women at certain times of the day. And there were significant negative correlations between myocardial repolarization markers and SDNN.

Heart rate variability is a noninvasive assessment method developed to measure cardiac autonomic functions. Disorders in HRV are closely associated with increased cardiovascular morbidity and mortality. It is suggested that women have different autonomic tone and control than men, which is the underlying cause of cardiovascular events that start in older ages and have less mortality and morbidity.

The autonomic nervous system plays a crucial role in the maintenance of cardiovascular hemostasis. It is understood from the studies that vagal tone decreases and sympathetic tone increases with increasing age.^17^ Investigating the effects of gender and daily fluctuations on the autonomic nervous system and investigating the relationship between cardiac repolarization markers will provide new information on autonomic physiology and provide insight into gender differences and daily changes on autonomic functions and cardiac repolarization. Gender differences in the functions of the autonomic nervous system and fluctuations at different times of the day will be effective in the progression of cardiac diseases such as coronary artery disease and heart failure, and mortality and morbidity outcomes.

Significant fluctuations in HRV and statistically significant reductions will be closely associated with increased cardiovascular mortality. This increase in cardiovascular mortality and morbidity will apply with or without underlying structural heart disease. It has been shown to be the underlying cause of increased cardiovascular mortality owing to decreases in HRV, increased sympathetic tone, and decreased vagal activity. Long‐term increased sympathetic activity and prolonged decreased vagal activity, with or without underlying heart disease, will eventually lead to mortality and morbidity.^3^

HRV is used to monitor physiological, pathological, and pharmacokinetic changes in the autonomic nervous system.^18^ Decreased HRV parameters are always closely associated with increased cardiovascular mortality.^19^ In previous studies, it was found that HRV parameters were higher in females and, therefore, vagal activity was higher in females than males.^20^ In our study, HF showing vagal tone was significantly higher in women than in men. In addition, LF, which is considered to exhibit sympathetic activity, was higher in men than in women. It is emphasized that the reason behind this is the estrogen hormone. In addition, estrogen has been shown to facilitate autonomic control over the heart in favor of vagal activity in women.^21^ Excessive sympathetic activity in healthy men compared with healthy women can be explained by the excess neuron in the sympathetic ganglia and the response to high muscular sympathetic activity.^22^, ^23^

Undesirable cardiovascular events are well known in the morning and occur in greater amounts and rates. Circadian changes in the autonomic nervous system may contribute to these events. Studies have shown that when the rates of unwanted cardiovascular events during the day are examined, higher rates of morbid and mortal cardiovascular events occur in the morning.^5^, ^24^

HRV peaks in the second half of the night in circadian rhythm.^25^ Increased vagal activity is the underlying cause of this peak of HRV.^26^ In the transition from morning sleep to wakefulness, HRV begins to reflect the effect of sympathetic activity by releasing parasympathetic activity parallel to the cortisol peak.^27^ Between the hours of 10.00 and 12.00 in the morning, the highest heart rates during the day are recorded, corresponding to the time of waking, and then begins to fall, and after a slight peak in the afternoon, the fall continues until night. As can be seen here, HR also follows a circadian rhythm. In addition, blood pressure, cardiac output, and serum catecholamine levels show a circadian variation.^28^, ^29^ In our study, women had the highest SDNN value at 07.00 am in the morning, and the lowest SDNN value at 01.00 am. In males, the highest SDNN value was again at 07.00 am, whereas the lowest SDNN value was at 01.00 pm.

Only HRV parameters do not reveal a circadian rhythm. In addition, malignant ventricular arrhythmias and sudden cardiac death follow a circadian rhythm. In particular, these conditions make a high peak immediately after waking up in the morning. These findings are based on the symptomatic onset of malignant ventricular arrhythmia detected in 24‐hour rhythm holter investigations and shocks in ICD patients.^30^, ^31^, ^32^ As a result, cardiac electrophysiology and malignant ventricular arrhythmias (sustained ventricular tachycardia and ventricular fibrillation) show a circadian rhythm and it is concluded that repolarization times reach the longest values of the day immediately after awakening and this may be effective in ventricular arrhythmogenesis.^33^

Electrophysiological studies have shown that the peak of the T wave coincides with the end of the repolarization of epicardial cells, while the end of the T wave coincides with the end of the repolarization of myocardial M cells. Delay in repolarization time from endocardial cells to epicardial cells, or in other words increases in repolarization time, has proven to be closely related to malignant ventricular arrhythmias.^34^ In one study it was found that there was an association between increased and delayed repolarization times with inducing or spontaneous, life‐threatening malignant ventricular arrhythmias in high‐risk ICD patients.^12^

In general, simple manual measurements of the T wave can be used for cardiovascular risk stratification both in patients with underlying structural heart disease and in the general healthy population.^11^ Therefore, the investigation of T‐wave morphology by simple methods has attracted much attention in the literature. In this regard, a number of simple methods have been developed for the duration, area, amplitude, and slope of the T wave.^12^

Nowadays, one of the most commonly used methods is the T peak‐end interval (Tp‐e), which is the distance from the peak of the T wave to the isoelectric line. Significant prolongations in Tp‐e (regional cardiac repolarization dispersion indicator) interval was closely associated with increased cardiovascular deaths.^13^, ^35^

Electrophysiological studies have shown that different repolarization patterns occur in men and women.^36^ In a study conducted on rabbits, J‐T peak and Tp‐e interval values were obtained showing different repolarization times in males and females.^9^ In our study, we found different repolarization markers with different values in healthy men and women at different times on the same day. And this difference was statistically significant.

Tp‐e interval, Tp‐e/QT, and Tp‐e/QTc are markers that indirectly show repolarization differences of the heart and, when increased, have been proven to be closely associated with malignant ventricular arrhythmias. These markers were found to be prolonged in many diseases in which malignant ventricular arrhythmia and sudden cardiac death rates were significantly increased, compared with the normal population and increased compared with the control group. Some of them are Brugada syndrome, coronary artery disease, heart failure^37^arrhythmogenic right ventricular cardiomyopathy,^38^ vasospastic angina,^39^ and liver cirrhosis.^40^

## 5 CONCLUSIONS

Variations in Tp‐e, cTp‐e interval, and Tp‐e/QT, Tp‐e/QTc, and cTp‐e/QTc ratios at different time intervals in a day have not been previously investigated in healthy individuals. In our study, we found that Tp‐e interval, cTp‐e interval, and Tp‐e/QT, Tp‐e/QTc, and cTp‐e/QTc ratios varied significantly throughout the day in both men and women. In addition, there were significant negative correlations between the Tp‐e interval and the SDNN values. These results will contribute to an understanding of the importance of pharmacochronotherapy.

## CONFLICT OF INTEREST

Authors declare no Conflict of Interest for this article.

## STUDY LIMITATIONS

In our study, there are relatively few cases. A greater number of case studies will contribute to obtaining more accurate information. In addition, a comparison of healthy males and females with malignant ventricular arrhythmias and the occurrence of malignant ventricular arrhythmias will contribute to obtaining more healthy data.
